# Sociodemographic, laboratory, image data and predictors of gravity risk in patients with COVID-19

**DOI:** 10.1371/journal.pone.0256331

**Published:** 2021-08-19

**Authors:** Víctor de Oliveira Costa, Eveline Montessi Nicolini, Bruna Malaquias Arguelles da Costa, Victor Hugo Perches Ferreira, Ana Julia Rodrigues Tonisi, Nathália Munck Machado, Marcos de Assis Moura, Jorge Montessi, Lincoln Eduardo Villela Vieira de Castro Ferreira, Rogério Leal Campos, Patricia Moreira Costa, Maria Antônia Campos

**Affiliations:** 1 Medicine, Faculdade de Ciências Médicas e da Saúde de Juiz de Fora – Suprema, Juiz de Fora, Minas Gerais, Brazil; 2 Physics, Universidade Federal de Juiz de Fora – UFJF, Juiz de Fora, Minas Gerais, Brazil; 3 Department of Toracic Surgery, Hospital Monte Sinai, Juiz de Fora, Minas Gerais, Brazil; 4 Nursing, Faculdade de Ciências Médicas e da Saúde de Juiz de Fora – Suprema, Juiz de Fora, Minas Gerais, Brazil; 5 Medicine, Universidade Federal de Juiz de Fora, Juiz de Fora, Minas Gerais, Brazil; 6 Department of Population Health, University of Kansas Medical Center, Kansas, United States of America; 7 Department of Infectology, Faculdade de Ciências Médicas e da Saúde de Juiz de Fora - Suprema and Universidade Federal de Juiz de Fora (UFJF), Juiz de Fora, Minas Gerais, Brazil; 8 Digestive Endoscopy Unit, Hospital Monte Sinai and Hospital Universitário – UFJF, Juiz de Fora, Minas Gerais, Brazil; 9 Department of Emergency, Hospital Monte Sinai, Juiz de Fora, Minas Gerais, Brazil; 10 Department of Intensive Care, Hospital Monte Sinai, Juiz de Fora, Minas Gerais, Brazil; Kaohsuing Medical University Hospital, TAIWAN

## Abstract

**Introduction:**

The effects, severity, and prognosis of COVID-19 infections do not follow a linear pattern in different locations, but change according to the epidemiological data and social issues in each region.

**Aims:**

The purpose of the current study is to provide the clinical and epidemiological standard of the population affected by COVID-19 in the city of Juiz de Fora, MG to better understand the disease and its risk factors, in order to enable more appropriate conduct for patients.

**Methods:**

A retrospective observational study was carried out from March to August of 2020, with 266 participants admitted to the emergency department of the Instituto de Clínicas e Cirurgia de Juiz de Fora—Hospital Monte Sinai. Data were tabulated, analyzed, and classified according to the outcome using an ordinal regression model.

**Results:**

Among the 266 admitted patients, the most common findings were ground-glass opacifications on chest CT (78.8%), cough (75.6%), fever (58.4%), and rhinorrhea (34.5%). There were greater severity and greater need for hospitalization and admission to the ICU in patients who were male, tachypneic at the time of admission, with older age, and with underlying diseases.

**Conclusion:**

Collected data allowed for a better understanding of the disease, its severity criteria, and its pattern of affection in Juiz de Fora, MG. More studies based on the analysis of the behavior of COVID-19 in different regions must be carried out, to improve treatment and support to local populations.

## Introduction

On March 11, 2020, a pandemic was declared by the World Health Organization (WHO) as a result of the outbreak of the emerging SARS-CoV-2. Infections first emerged in the Chinese city of Wuhan, in the Hubei province, as pneumonia of unidentified cause, receiving global attention due to the fast contagious evolution of the disease [1]. By September of 2020, the number of cases reported worldwide already exceeds 102 million, with more than 2 million confirmed deaths [2].

Due to the rapid spread of the disease, several countries are publishing new epidemiological information daily [3]. According to the epidemiological bulletins, Brazil accounts for a total of 3,950,931 confirmed cases [4], 218,781 of which were reported in the state of Minas Gerais (MG) [5]. In the municipality of Juiz de Fora, the disease scenario shows 4,873 confirmed cases and 146 confirmed deaths by COVID-19 [6].

In view of the global level of infection, knowledge of clinical aspects and development of the disease is of utmost importance. This study aims to describe the epidemiological and clinical characteristics of patients with confirmed SARS-CoV-2 infection, diagnosed and treated at a hospital in Juiz de Fora, MG.

## Methods

### Design, ethical aspects, and procedures

This is a retrospective observational study. Data were collected through electronic medical records carried out by the researchers, which were conducted at the Instituto de Clínicas e Cirurgia de Juiz de Fora—Hospital Monte Sinai. This hospital covers the Zona da Mata region of Minas Gerais, which has a population of approximately 2 million. We performed data collection between March and August of 2020 and research stages began only after approval by the Research Ethics Committee (Approval No. 4,080,157, Santa Casa de Misericórdia de Juiz de Fora / MG). After collecting information from electronic medical records, data were tabulated using Google Forms. We then proceeded by correcting typing errors before conducting data analysis.

### Participants

All patients who were in the emergency department with COVID-19 symptoms were part of the research. The inclusion criteria were: 1) Patients of all ages, 2) non-pregnant, and 3) COVID-19 confirmed by RT-PCR laboratory tests. Patients that did not show positivity for RT-PCR laboratory tests and/or rapid test for COVID-19 were excluded. Also, patients with poorly written medical records (e.g., loss of data, such as vital signs on admission, comorbidities, and lack of detail regarding the patient’s previous symptoms) could not be part of the sample. Therefore, we excluded participants who had more than two missing data in the records. In the end, 266 participants composed the final sample.

### Data detailing

Data were collected and stored in electronic medical records within the hospital’s computer system. Data from the physical examination performed by the doctors such as the patients´ vital signs were also stored in the electronic medical record. Regarding radiography and computed tomography, we opted for the latter most of the time, due to the hospital´s protocols, being performed at the time of the patient’s admission, hence the low number of radiographs collected. Data such as symptoms before hospitalization, presence of fever, and contact with sick persons were collected using a patient’s self-report questionnaire, that was stored in the hospital’s electronic medical record. In some points, there was a loss of data, such as the question regarding if the patient had contact with a sick person. In this situation, a smaller number of analyzed patients will be seen. As stated above, only medical records in which a variety of information was missing were excluded. Therefore, the lack of only some specific information did not fit the exclusion criterion. Consequently, in some fields, the absence of these data will be evident, and there will not be a total of 266 participants.

### Data analysis

After tabulation, we performed an exploratory and inferential analysis of the data. Descriptive analysis was made using frequencies, percentages, measures of central tendency, and dispersion. All analyses were performed using R Core Team version 4.0.2 (2020) and SPSS version 20.0.0 [7, 8].

To identify variables associated with hospitalization and ICU admission, we used an ordinal regression model. This approach provided advantages over other generalized linear models, which may oversimplify data by assuming equal intervals between response categories. Instead, ordinal regression treats a dependent variable as pairwise, ordered groupings of consecutive nominal elements. The ordinal regression captures the odds of moving up 1 level in the ordering under the proportional odds assumption across multiple levels. The dependent was hospitalization, with three levels: 1) not admitted or discharged, 2) hospitalized in a regular ward, and 3) admitted to the ICU. The covariables used for the model were: sex, age, systemic arterial hypertension, diabetes, asthma, coronary disease, fever, heart rate, respiratory rate, and patient characteristics, such as if the patient was a health professional and/or reported having contact with a sick person. We excluded 45 cases due to missing data regarding age and patients´ characteristics, and the final model had 221 participants. This analysis was conducted using R and the MASS and Brant packages [7, 9, 10]. Adjusted odds ratio (OR) and 95% confidence intervals (CI) were calculated, and the Brant test was used to verify the OR assumptions and proportionality. We also calculated the generalized variance inflation factor (GVIF) to confirm the absence of any substantial multicollinearity. The GVIF was used due to the nominal nature of some independent variables.

## Results

### Characteristics of the sample

Most patients were male (57.9%) and had a mean age of 44.71 (SD 16.83). Regarding race, 149 (79.7%) were white, 13 (7%) were black and 25 (13.4%) were brown.

In the sample, 127 patients reported having contact with people infected with COVID-19, 97 received influenza vaccine, and a total of 50 patients were health professionals. The most prevalent comorbidities were Systemic Arterial Hypertension, with a total of 72 patients (27.1%), followed by heart disease in 16 (6.0%) patients. The number of comorbidities per patient was also assessed: 158 (59.4%) had no comorbidities, 66 (24.8%) had only 1 comorbidity, 24 (9.0%) had 2 comorbidities, and 18 (6.8%) had 3 or more comorbidities (Table 1).

**Table 1:** Epidemiological data of the sample separated by outcome.

	Not admitted	Hospital room	ICU	Total
Gender [Male]	90(58.4)	34(22.1)	30(19.5)	154(100.0)
Age				
0–9	2(1.2)	1(1.7)	0(0.0)	3(1.1)
10–19	3(1.8)	1(1.7)	0(0.0)	4(1.5)
20–29	31(18.3)	4(6.9)	0(0.0)	35(13.2)
30–39	64(37.9)	12(20.7)	6(15.4)	82(30.8)
40–49	32(18.9)	14(24.1)	3(7.7)	49(18.4)
50–59	22(13.0)	13(22.4)	4(10.3)	39(14.7)
60–69	14(8.3)	8(13.8)	13(33.3)	35(13.2)
70+	1(0.6)	5(8.6)	13(33.3)	19(7.1)
Total	169(100.0)	58(100.0)	39(100.0)	266(100.0)
Race				
White	83(74.8)	39(84.8)	27(90.0)	149(79.7)
Black	11(9.9)	1(2.2)	1(3.3)	13(7.0)
Brown-skinned	17(15.3)	6(13.0)	2(6.7)	25(13.4)
Total	111 (100.0)	46(100.0)	30(100.0)	187(100.0)
Health professional [Yes]	35(70.0)	13(26.0)	2(4.0)	50(100.0)
Health professional [No]	44(20.6)	134(62.6)	36(16.8)	214(100.0)
Previous contact with COVID [Yes]	98(77.2)	22(17.3)	7(5.5)	127(100.0)
Previous contact with COVID [No]	29(28.2)	54(52.4)	20(19.4)	103(100.0)
Received influenza vaccine [Yes]	64(66.0)	21(21.6)	12(12.4)	97(100.0)
Received influenza vaccine [No]	19(18.3)	76(73.1)	9(8.6)	104(100.0)
Comorbidities				
SAH	29(64.4)	20(64.5)	23(59.0)	72(27.1)
Diabetes mellitus	3(6.7)	4(12.9)	1(2.6)	8(3.0)
Immunosuppressive Condition	4(8.9)	2(6.5)	1(2.6)	7(2.6)
Dyslipidemia	3(6.7)	0(0.0)	1(2.6)	4(1.5)
Heart disease	4(8.9)	4(12.9)	8(20.5)	16(6.0)
Asthma	2(4.4)	1(3.2)	0(0.0)	3(1.1)
COPD	0(0.0)	0(0.0)	5(12.8)	5(1.9)
	45(100.0)	31(100.0)	39(100.0)	115(100.0)
Number of comorbidities				
0	120(71.0)	27(46.6)	11(28.2)	158(59.4)
1	36(21.3)	19(32.8)	11(28.2)	66(24.8)
2	10(5.9)	7(12.1)	7(17.9)	24(9.0)
3+	3(1.8)	5(8.6)	10(25.6)	18(6.8)
Total analyzed	**169(63.5)**	**58(21.8)**	**39(14.7)**	**266(100.0)**

Furthermore, we also analyzed patients’ vital signs during emergency room admission. Twenty-two (8.4%) had a fever between 37.5–38.5 degrees Celsius, and 26 (9.9%) were in a feverish state between 37 to 37.5 degrees. In 45 (17.2%) of the patients, tachycardia was noted at the time of admission, and 35 (13.4%) had tachypnea. Besides, we performed chest CT in 208 patients (78.8%) and found ground-glass opacifications in 149 (71.6%). The presence of non-calcified nodules of an inflammatory character was noted in 30 (14.4%) patients and pleural effusion in 5 (2.4%). Chest radiography was performed in only 7 (2.7%) patients. We also assessed which medications the patients were using when they arrived at the emergency room. Seventy-seven (28.9%) reported using some type of medication, with the most common being anti-flu drugs such as antihistamines and analgesics, as seen in 52 patients (67.5%). Hydroxychloroquine use was reported in 7 (9.1%) patients, Azithromycin in 25 (32.5%), and Ivermectin in 7 (9.1%), as shown in Table 2.

**Table 2:** Physical and initial examination of the patient on admission to the emergency department.

Assessment of vital signs of patients on admission		
Axillary Temperature	N	%
35.5–37°C (normal)	212	80.9
37–37.5°C (feverish)	26	9.9
37.5–38.5°C (moderate fever)	22	8.4
>38.5°C (elevated fever)	2	0.8
Total	262	100
Heart Rate		
60–100 (normal)	216	82.8
> 100 (tachycardia)	45	17.2
Total	261	100
Respiratory Rate		
< 14 (bradypnea)	3	1.1
14–20 (eupnea)	223	85.4
> 20 (tachypnea)	35	13.4
Total	261	100
Image exam at admission		
**Computer Tomography (N = 264)**		
**Performed**	208	78.8
Ground-glass opacifications	149	71.6
Nodules	30	14.4
Pleural effusion	5	2.4
Normal	53	25.5
**Not performed**	56	21.2
**Chest X-ray (N = 264)**		
**Performed**	7	2.7
Infiltrado bilateral	3	42.9
Normal	4	57.1
**Not performed**	257	97.3
Medication use at admission		
Yes	77	28.9
Anti-flu drugs	52	67.5
Hydroxychloroquine	7	9.1
Azithromycin	25	32.5
Sulfamethoxazole + Trimethoprim	1	1.3
Amoxicillin + clavulanate	3	3.9
Systemic corticosteroids	3	3.9
Enoxaparin	3	3.9
Ivermectin	7	9.1
No	189	71.1

N: Number of patients

Regarding symptoms, 199 (75.6%) of patients had cough, 63 (24%) dyspnoea, 69 (26.1%) odynophagia, 12 (4.6%) productive sputum, 89 (34%) headache, 154 (58.8%) fever or feverish condition, 91 (34.5%) rhinorrhea, 53 (20.2%) diarrhea, and 4 (1.5%) were totally asymptomatic. Patients who reported fever at home had a mean temperature of 38.2 Celsius degrees (SD 0.53) and a maximum of 40 degrees. The duration of symptoms is presented in Table 3.

**Table 3:** Time of symptoms and prevalence collected on admission to the emergency department.

Duration of symptoms										
Days	01 a 04	%	05 a 08	%	09 a 12	%	12+	%	DHS	%
Cough	108	41.1	70	26.6	8	3	13	4.9	64	24.3
Dyspnoea	44	16.7	16	6.1	2	0.8	1	0.4	200	76
Odynophagia	39	14.8	23	8.7	4	1.5	3	1.1	194	73.8
Productive Sputum	9	3.4	1	0.4	1	0.4	1	0.4	252	95.8
Headache	53	20.3	30	11.5	3	1.1	3	1.1	172	65.9
Fever / Feverish state	97	37	46	17.6	8	3.1	3	1.1	108	41.2
Rhinorrhea	44	16.7	38	14.4	6	2.3	3	1.1	172	65.4
Diarrhea	35	13.3	12	4.6	2	0.8	4	1.5	210	78.9
Total of patients by symptom										
Cough	199	75.6				Mean	SD	Min.	Max.	
Dyspnoea	63	24			Fever	38.20	0.53	37.3	40	
Odynophagia	69	26.1								
Productive Sputum	12	4.6								
Headache	89	34								
Fever / Feverish state	154	58.8								
Rhinorrhea	91	34.5								
Diarrhea	53	20.2								
Asymptomatic	4	1.5								
Total analyzed	263									

SD = Standard Deviation; DHS = Did not have this symptom

### Characteristic trends across outcome groups

The sample was separated according to the outcome of the patient: not admitted, admitted to the ward, and admitted to the ICU. A total of 50 (18.9%) health professionals were part of the sample, with only 2 (4.0%) being admitted to the ICU. The data “Previous contact with COVID” and “Received influenza vaccine” were not filled out by some patients, so they do not add up to 266 at the end. However, this was not a patient exclusion factor. Of 230 patients analyzed, 127 (55.2%) reported having had contact with a person who had symptoms, of whom 22 (17.3%) were admitted to the hospital ward and 7 (5.5%) to the ICU. Ninety-seven patients (48.3%) had received the influenza vaccine in the last campaign. Of these, 21 (21.6%) needed hospital admission, and 12 (12.4%) were referred to the ICU. The distribution of these data can be seen in Table 1.

**Fig 1:**
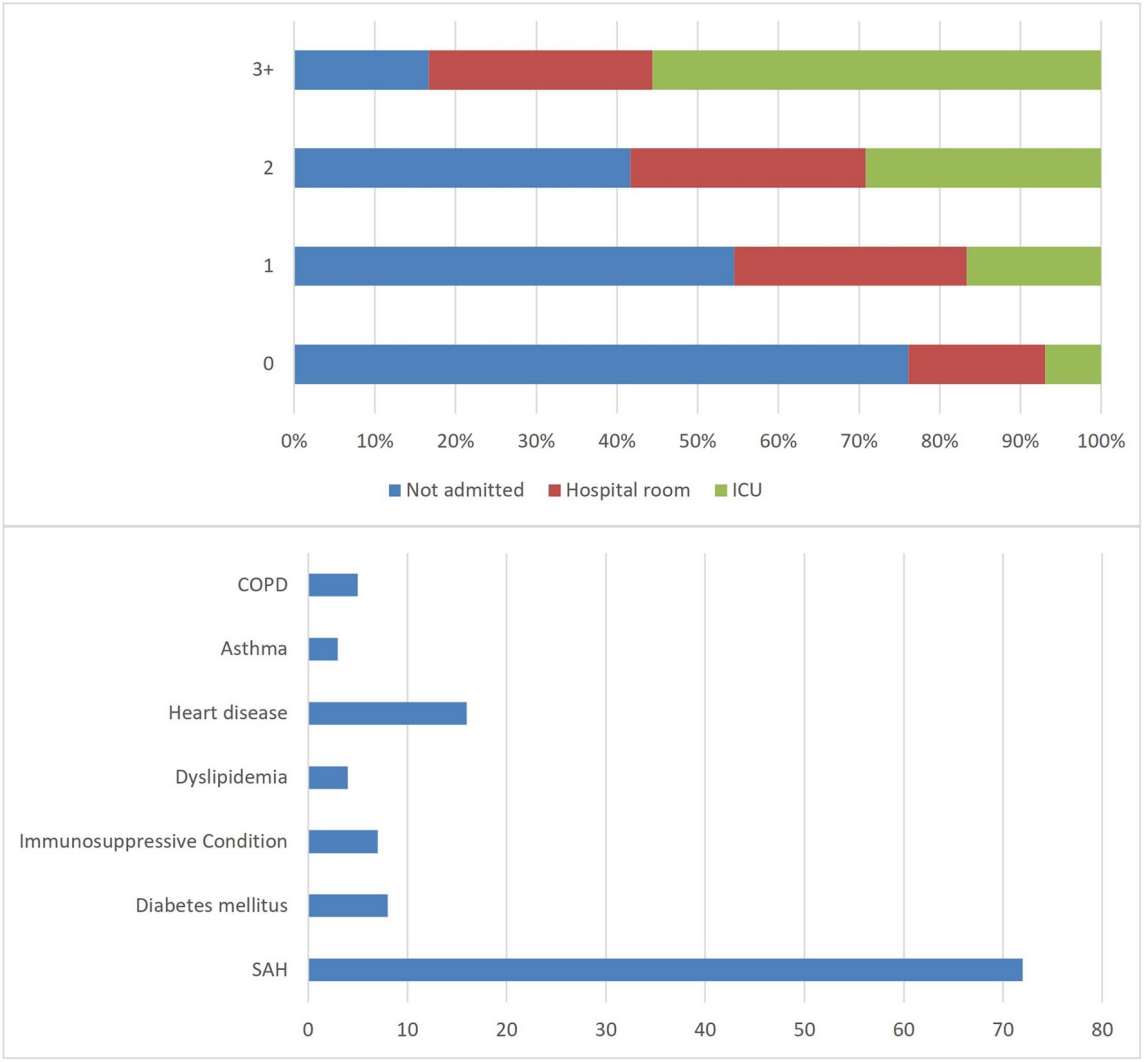
Distribution of comorbidities by area of hospitalization and its prevalence.

Of the total of 266 patients, 169 (63.5%) were not admitted—that is, they passed through the emergency room and returned home. Fifty-eight (21.8%) went to non-ICU hospitalization beds, and 39 (14.7%) went to the ICU. The comorbidities studies are shown in Table 1. The most prevalent comorbidity was Systemic Arterial Hypertension (SAH), which was present in 27.1% of all cases, and in 59% of patients admitted to the ICU. Heart diseases were present in 20.5% of all cases and COPD, 12.8%. We distributed the number of comorbidities in each patient by area of hospitalization, as verified at the top of Fig 1. Notably, patients with more comorbidities represent a larger proportion of those admitted to the ICU. In Fig 1, a column chart was placed to demonstrate the prevalence of comorbidities in the studied population.

**Fig 2:**
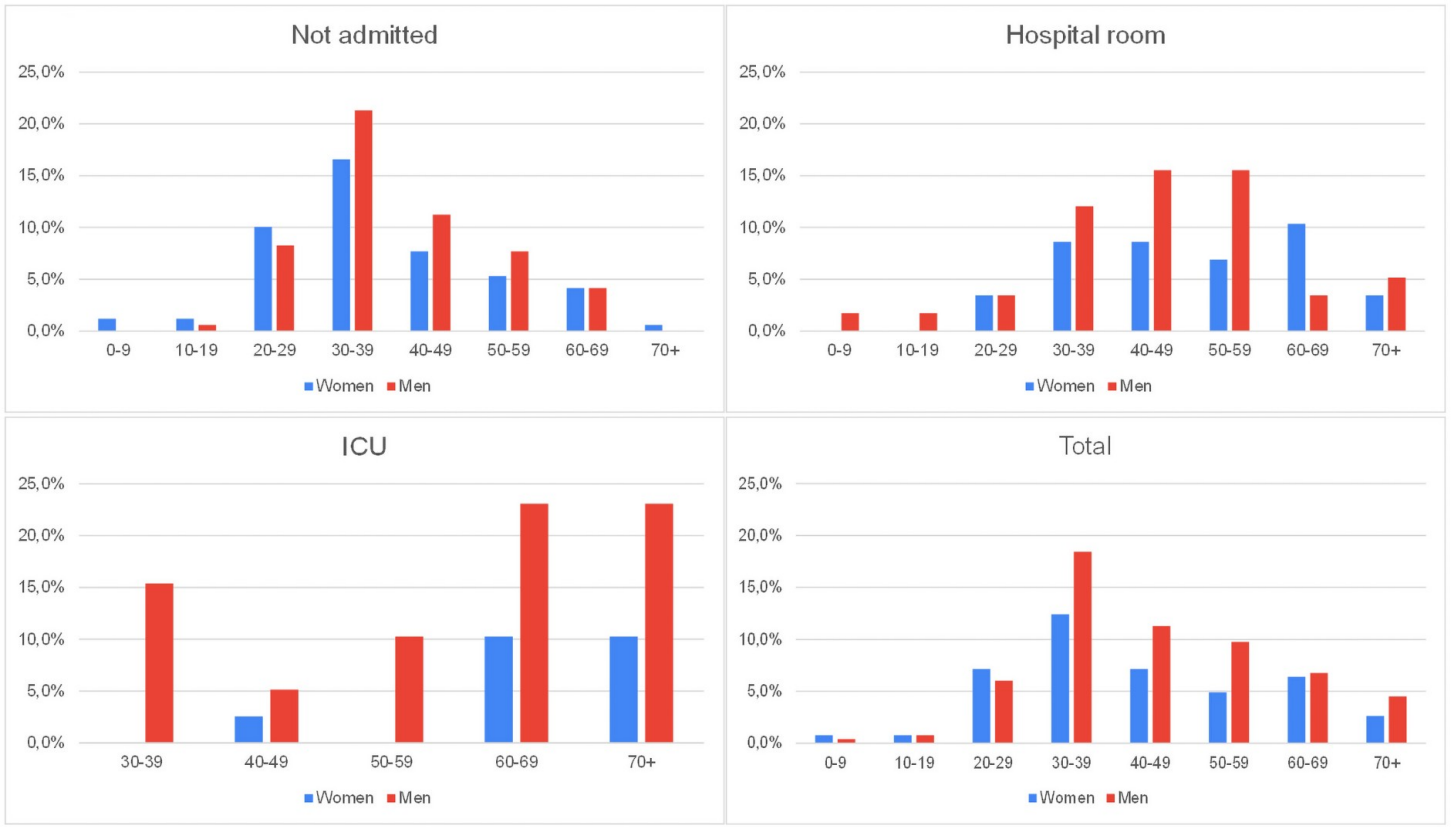
Distribution of patients according to age, sex, and outcome.

Also, for a better understanding of the studied population, patients were separated into groups by age. Their prevalence in each environment was also verified, as shown in Table 1. We found that patients over 50 years of age corresponded to 30 (76.9%) of all patients admitted to the ICU. This age group also accounts for 26 (44.8%) of those that needed hospitalization and for only 37 (21.9%) of the non-hospitalized patients. In Fig 2, the charts with the percentage distribution of patients are designed according to gender and patient outcome.

### Results from ordinal regression

To determine predictors of hospitalization, we performed an ordinal logistic regression with complete data from 221 participants. After adjusting for all key covariates, we found that being male (OR 1.85, p = 0.09) and the presence of tachypnea (OR 20.75, 95% CI 6.94–61.98, p = 0.01) were associated with an increased risk of being hospitalized in a regular ward or admitted to the ICU. Being older was also a predictor of hospitalization. Patients aged 70 years or older (OR 35.95, p = 0.001) were more likely to be hospitalized in a regular ward and admitted to the ICU, followed by patients between 60 and 70 years old (OR 5.91, p = 0.02). Finally, patients who reported having had contact with a person with COVID-19 were associated with a protective factor for hospitalization (OR 0.33, p = 0.002). The results from ordinal regression are presented in Table 4.

**Table 4:** Results from ordinal regression of characteristics associated with hospitalization with three levels of response (N = 221).

Variable		Odds ratio*	p-value*	X2**	Probability**
Hospitalization (*intercepts*)					
	Discharged | Hospitalization		0.0007		
	Hospitalization | ICU		< .0001		
Sex	Female	Reference			
	Male	1.85 (0.89–3.77)	0.0982	2.74	0.1
Age	(20,30]	Reference			
	(30,40]	1.66 (0.45–6.17)	0.4512	2.73	0.1
	(40,50]	3.22 (0.87–11.94)	0.0797	6.27	0.01
	(50,60]	2.00 (0.41–9.69)	0.3903	4.19	0.04
	(60,70]	5.91 (1.27–27.59)	0.0237	0.39	0.53
	(70,100]	35.95 (4.68–276.23)	0.0005	0	0.99
Systemic Arterial Hypertension	No	Reference			
	Yes	1.25 (0.46–3.38)	0.6615	1.11	0.29
Diabetes	No	Reference			
	Yes	1.60 (0.28–9.19)	0.5979	0.31	0.58
Asthma	No	Reference			
	Yes	3.58 (0.29–43.87)	0.3182	0	0.99
Coronary Disease	No	Reference			
	Yes	1.34 (0.31–5.84)	0.7003	0.21	0.64
Fever	Normal (35.5–37°C)	Reference			
	Febrile (37–37.5°C)	1.75 (0.66–4.63)	0.2598	0.66	0.42
	Fever (> 37.5°C)	1.88 (0.62–5.65)	0.2620	1	0.35
Heart Rate	60–100 (Normal)	Reference			
	> 100 (Tachycardia)	1.92 (0.77–4.80)	0.1625	1	0.07
Respiratory Rate	14–20 (Eupnea)	Reference			
	> 20 (Tachypnea)	20.75 (6.94–61.98)	< .0001	1	0.37
Patient is a healthcare professional	No	Reference			
	Yes	1.83 (0.78–4.30)	0.1682	1	0.4
Paciente had contact with a person with COVID-19	No	Reference			
	Yes	0.33 (0.16–0.67)	0.0022	0.3	0.58

*OR and p-value from ordinal regression analysis.

**X2 and probability from the Brant test.

## Discussion

Among patients admitted with COVID-19 infection, 36,5% required hospitalization, a rate lower than that found in other studies, such as the publication by Rivera-Izquierdo M et al. [11], in which the value obtained was 48,7%. This difference can be explained by the lower average age and number of comorbidities per patient in our study. Age is a well-established risk factor for hospitalization by SARS–COVID, especially among the elderly population (> 60 years), with a relative increase in the Odds Ratio for higher ages [11–14]. Among our patients, the mean age was 44.71, a lower value than that found in studies conducted outside Brazil, where the elderly population is proportionally more significant. Furthermore, multivariate analysis showed a higher chance of hospitalization for older ages.

According to a meta-analysis published by the European Journal of Epidemiology, although the mortality rate of infection is higher in the elderly and infirm patients, middle-aged adults are also at risk: the mortality rate for this group, when infected, is two times greater than the annualized risk of a fatal automobile accident, and much greater than the risk of death by seasonal flu. Consequently, public health measures should also be targeted at these groups to reduce the incidence of mortality [15].

Concerning gender, our study showed that greater severity and need for hospitalization or admission to the ICU were seen in males. A meta-analysis published by Nature evidenced that although there were no differences between males and females regarding the number of confirmed cases, male patients were almost three times more likely to require intensive care or to die. The explanation for this is multifactorial, involving female estrogen protection and greater innate and adaptive immune response (higher number of CD4+ T cells and more robust CD8+ T cell cytotoxic activity, along with a higher production of immunoglobulin by B cells) [16, 17].

Underlying diseases increase the risk of affection and hospitalization by COVID-19, in addition to being important indicators of severity. Among them, systemic arterial hypertension, cardiovascular disease, dyslipidemia, and diabetes mellitus stand out, followed by COPD, CKD, and immunosuppression (HIV, transplants) [11–13, 18–21]. Hypertension occupies the most prominent place and has received the most attention in recent studies, with values ranging from 4,5% in non-critically ill individuals [22] up to >50% in hospitalized groups [11, 13]. It is important to highlight that the majority of studies showed a prevalence of hypertension between 15 to 35%, especially in China [23–29]. In theory, asthmatic patients would be more susceptible to SARS-CoV-2 infection, but studies have not shown the expected prevalence of patients with this condition among patients with COVID-19 [30]. Concerning our patients, 27.1% had SAH, a value similar to those found in Chinese studies. 6% had heart disease, 3% DM, 2.6% immunosuppression, 1.9% COPD, 1.5% dyslipidemia and 1.1% asthma.

Recent publications show that men (60.3%) and those who had contact with individuals known to be infected with COVID-19 had a higher risk of testing positive for the disease [12, 13]. Knowing that the main transmission mechanism of SARS-CoV-2 is through infected respiratory droplets, either by direct or indirect contact with the conjunctival nasal mucosa, this finding becomes plausible since most infections occur through close contacts, such as talking to an infected person for 15 minutes, within 2 meters [31]. Our study observed a correlation similar to the one exposed above: 57.9% of the admitted patients were male and 55.2% of the patients claimed to have had contact with a sick person, with 63.2% of these claiming that the contact occurred in their workplace.

Regarding race, in a systematic review recently published by The Lancet, black individuals were twice as likely to be infected with SARS-CoV-2 when compared to white individuals. The explanation for this finding is multifactorial and mainly includes issues related to lower socioeconomic levels among the black population, such as a higher probability of living in overcrowded homes and essential employment (less possibility of working from home) [32]. Among our patients, 79.7% of those who required hospitalization were white. This value may be considered biased since the hospital where the study was conducted is a private institution, whose access is limited to a population with better socioeconomic conditions, which is mostly white in the region.

Health professionals have been highly exposed and affected by the disease. Most recent studies show a higher risk of SARS-CoV-2 infection among healthcare professionals, especially those on the front lines. Lack of or inadequate use of personal protective equipment (PPE), contact with colleagues or patients in the early stages of unsuspected infections, with high viral loads, are the main causes that lead to increased risk in this population [33, 34]. In China, 3,300 had been infected and, in Italy, 20% tested positive for the disease at the beginning of March [35]. In our sample, 18.9% of the admitted patients were health professionals. Furthermore, being a healthcare professional did not denote a chance of hospitalization, but patients who claimed to have had contact with a sick individual, with the health professionals participating in this group, proved to have a protective factor for hospitalization, which may suggest the formation of prior immunity, leading to them not requiring more specialized treatment. However, it is worth remembering that the study was conducted in only one hospital and there may be limitations in this result.

Important changes related to vital signs have been documented during the admission of patients infected with COVID-19, such as an increase in baseline temperature as well as heart and respiratory rates. This assessment is indispensable, since reduced SPO_2_ and increased blood pressure, heart rate, and respiratory rate are independent risk factors for mortality in patients with COVID -19, with emphasis on SPO_2_ and high blood pressure [36, 37]. A study published in The Journal of the American Medical Association (JAMA) revealed that at the time of screening, 30.7% of patients were febrile and 17.3% tachypneic [13]. Our study found an important agreement: 19.1% febrile and 13.4% tachypneic, with tachypnea being a significant parameter to increase the chance of hospitalization.

In a review by Pascarella G et al., the most prevalent symptoms found were fever, which affected more than 80% of cases, dyspnea, and cough, followed by other symptoms such as rhinorrhea, headache, diarrhea, nausea, vomiting, and myalgia [20]. In agreement with this study, a meta-analysis later published by the Journal of Medical Virology found that fever was the most common symptom (80.4%), followed by cough (63.1%) [17]. In our sample, the most common symptoms were similar, but unlike the referred meta-analysis, cough (75.6%) was more frequent than fever (58.8%) in our study. It is important to advise the patient that when fluid replacement is performed correctly, fever is usually self-limited and rarely indicates a serious problem. It is a tool to fight the virus and inhibit its replication [38]. Cough in SARS-CoV-2 is usually persistent, can be either dry or productive, and has a multifactorial etiology. The likely mechanisms that cause coughing involve inflammation, epithelial damage, mucoid impaction, and neuromodulatory changes [39].

To date, there are no medications that have been proven effective in curing COVID-19 [40]. Studies in this direction are being developed, with divergent results. In our study, 28.9% of patients were using medication at the time of admission. The main type consisted of anti-flu drugs, such as antihistamines and analgesics (67.5%). Among these medications, Paracetamol is recommended, due to the lower likelihood of adverse effects [41]. Furthermore, Paracetamol can help reduce fever, but it is important to note that maintaining a mild fever can be a favorable tool in fighting the virus [38]. 32.5% of the patients used Azithromycin and 9.1% Hydroxychloroquine. Both have been tested in association in a series of clinical trials, due to a possible acceleration of the virus eradication process, but more studies are needed [41]. Tests with isolated Hydroxychloroquine showed apparent efficacy and safety against COVID-19 associated with pneumonia [42]. Another 9.1% of patients used Ivermectin, which has shown benefits *in vitro* tests, but not *in vivo* when it comes to the treatment of COVID-19 [41]. Regarding Ivermectine, while the effectiveness of *in vivo* response still requires more research, it is already known that it can have a positive influence in some therapeutic options, such as reducing side effects related to Hydroxychloroquine use, such as QT prolongation, myopathy, and neuropathy [43]. Other medications less used at the time of admission were Amoxicillin + Clavulanate, Systemic Corticosteroids and Enoxaparin, each corresponding to 3.9%, and finally, Sulfamethoxazole + Trimethoprim, used by 1 patient (1.3%). These medications are less common in the literature and also have no evidence of efficacy to date.

Although a cure for COVID-19 has not been discovered so far, some drugs have recently shown significant results, reducing the chance of greater disease severity. Whatsmore, some can also be used as prophylactic and adjuvant therapy to COVID-19. Examples of these are vitamin D, zinc, and probiotics. Vitamin D inhibits the production of pro-inflammatory cytokines and increases those with anti-inflammatory properties. Thus, vitamin D deficiency is related to a greater intensity of inflammatory response, which increases morbidity and mortality in Sars-Cov-2 infection [44]. Zinc, in turn, has shown to be effective in reducing susceptibility to pathogens [45]. Probiotics increase immunological activity and help eliminate or reduce infections related to the respiratory tract, helping to balance the pulmonary microbiota [46]. Unfortunately, these studies are recent, and questioning the use of vitamin D, zinc, and probiotics at the time of admission was not part of our study.

## Conclusion

The present study evaluates several epidemiological data referring to COVID-19 infection, whose knowledge is essential in Brazil, where there is less evidence regarding the infection in populations from different regions. In addition, data inferences in this study may suggest risk factors for a greater chance of hospitalization, e.g., characteristics that denote a patient’s severity of illness.

Thus, it is of utmost importance to carry out more studies of this nature so that it is possible to assess viral behavior in different places in the world, in order to allow greater knowledge of the disease and the creation of more effective measures for treatment.

## Supporting information

S1 Appendix Data used for analysis in English. (XLSX)

S2 Appendix Data used for analysis in Portuguese. (XLSX)
